# Risk factors for intrafamilial spread of hepatitis B in northeastern Bosnia and Herzegovina

**DOI:** 10.4103/0256-4947.51821

**Published:** 2009

**Authors:** Nermin N. Salkic, Enver Zerem, Muharem Zildzic, Sead Ahmetagic, Elmir Cickusic, Farid Ljuca

**Affiliations:** a From the Department of Gastroenterology, Internal Medicine Hospital, University Clinical Center Tuzla, Bosnia and Herzegovina; b From the Infective Diseases Hospital, University Clinical Center Tuzla, Bosnia and Herzegovina; c From the Department of Laboratory Diagnostics, University Clinical Center Tuzla and Bosnia and Herzegovina; d From the Faculty of Medicine, University of Tuzla, Tuzla, Bosnia and Herzegovina

## Abstract

**BACKGROUND:**

Accurate estimations of hepatitis B virus transmission risk for any region in Bosnia and Herzegovina are not clearly established. We aimed to determine levels of risk associated with intrafamilial transmission of hepatitis B infection within families in our region.

**PATIENTS AND METHODS:**

Family members of 81 chronic carriers of hepatitis B surface antigen (>6 months positive and considered as index case) were tested for hepatitis B markers. For family members, we recorded their age, sex, and family relationship to the index case, and vaccination status.

**RESULTS:**

The proportion of HBsAg positive family members was 25/207 (12.1%), while the proportion of family members with evidence of exposure to HBV was 80/207 (38.6%). Only 17/207 (8.2%) family members had evidence of past HBV vaccination. Age was found to be a significant predictor of HBV exposure of family members (odds ratio 1.05, 95% CI 1.03-1.07, *P*<.001). In a multivariate analysis, HBsAg positivity was associated with a female index case (odds ratio 11.31, 95% CI 3.73-34.32, *P*<.001), HBeAg positivity in the index case (odds ratio 5.56, 95% CI 1.80-17.23, P<.005) and being a mother of the index case (odds ratio 9.82, 95% CI 2.43-39.68, *P*<.005). A female index case (odds ratio 4.87, 95% CI 2.21-10.72, *P*<.001), HBeAg positivity in the index case (odds ratio 3.22, 95% CI 1.15-9.00, *P*<.05) and being a mother of the index case (odds ratio 3.72, 95% CI 1.19-11.64, *P*<.05) were also risk factors for HBV exposure among family members. The combination of HBeAg positivity and female index case was a significant predictor for HBsAg positivity of family members (odds ratio 70.39, 95% CI 8.20-604.61, *P*<.001).

**CONCLUSIONS:**

Children of HBeAg positive mothers are at highest risk for becoming chronic carriers themselves and generally, the combination of female sex and HBeAg positivity dramatically increases the chances of HBV transmission within the family.

The World Health Organization (WHO) divides the world into areas of low, intermediate ate and high endemicity for hepatitis B virus (HBV) infection. Bosnia and Herzegovina, according to WHO estimates and published reports, is in the area of intermediate endemicity for HBV infection, with an HBsAg prevalence of 3.6% among first-time blood donors.^1^ Transmission of HBV within family or household contacts is not a rare phenomenon and family members of chronic HBV carriers are a well-established risk group.^2^–^8^ Although the precise means of HBV spread within families is not well defined, there are several risk factors associated with it.^4^,^5^,^7^ Several studies have evaluated routes of intrafamilial transmission of HBV in our region, and the difference in HBV exposure between family members of chronic HBV carriers and first-time blood donors.^3^,^7^–^9^ However, the true levels of risk associated with facilitated HBV spread within the family have not been analyzed in detail. We conducted a hospital-based study with the intention to analyze levels of risk for risk factors associated with intrafamilial transmission of hepatitis B infection within families in our region, thus determining the pattern of risk factors that eventually results in more HBV-positive family members of chronic hepatitis B patients.

## PATIENTS AND METHODS

The study was conducted prospectively during a 2-year period (2004-2006) at the Tuzla University Hospital, Bosnia and Herzegovina, and was approved by the University Clinical Center Tuzla Ethical Committee. Index cases were 81 consecutive patients that satisfied the criteria of being positive for HBsAg for a minimum of 6 months and were the first detected chronic HBsAg carriers within their families. They were tested for HBsAg and hepatitis B e antigen (HBeAg) with the ELISA test (Abbott Diagnostics, Wiesbaden, Germany), and their age and sex were noted. We defined chronic HBsAg carriers as those positive for HBsAg for at least a 6-month period. Exposure to HBV was defined as positivity for any HBV marker in the absence of HBV vaccination evidence. We offered HBV serology testing to family members of all chronic HBsAg carriers living in the same household. Two hundred and seven family members of index cases were tested for hepatitis B markers (HBsAg, HBeAg, anti-HBsAg, anti-HBcAg, anti-HBeAg) and their age, sex, family relationship to the index case and vaccination status were noted.

Statistical tests were performed using SPSS 15.0 (SPSS Inc, Chicago, IL, USA). Index cases were stratified by age, sex, and presence of HBeAg, while family members were stratified by sex, age, presence of HBV markers, relationship to index case, size of their corresponding family and vaccination status. Standard tests for descriptive statistics were used for determination of baseline characteristics of groups. Between-group differences in frequencies were investigated by the chi-square test. The Spearman rank-order correlation was used for determining correlation between age, HBV exposure and HBsAg seropositivity. Univariate logistic regression was used to determine factors associated with HBsAg positivity or HBV exposure. Factors found to be significant in univariate analysis were tested using stepwise multivariate regression analysis. All tests were performed with a 95% statistical significance level.

## RESULTS

Of 81 chronic HBV carriers there were 54 (66.7%) males and 27 (33.3%) females, with mean (SD) for age of 40 (12) years (range, 5 to 65 years). We detected positivity for HBeAg in 14/81 (17.3%), while the remaining 67/81 (82.7%) were HBeAg seronegative. There were 92/207 (44.4%) males and 115/207 (55.6%) females in family members of index cases, including 13 fathers, 16 mothers, 56 sons, 56 daughters, 4 brothers, 3 sisters, 16 husbands and 43 wives. Mean (SD) for age in this group was 28 (16) years (range, 2 to 66 years). Only 17/207 (8.2%) family members had evidence of past HBV vaccination. There were 8/207 (3.9%) family members younger than 5 years, and 6/8 (75%) had evidence of previous vaccination.

The proportion of HBsAg-positive family members was 25/207 (12.1%), while the proportion of family members with evidence of exposure to HBV was 80/207 (38.6%). The highest proportion of HBsAg-positive family members was found among parents of index cases, while the highest ratio of HBV exposure was found within siblings and parents, respectively (Table 1). For family members whose index case was a female there were significantly more HBsAg positives than among those whose index case was a male (Table 2). Correspondingly, there were significantly more family members with evidence of HBV exposure among those with a female index case than among those with a male index case. Family members of HBeAg positive index cases (10/28; 35.7%) had a higher proportion of HBsAg positives (χ^2^=14.56; *P*<.001) in comparison to family members of HBeAg negative index cases (15/179; 8.4%). The proportion of HBV exposed was also higher (χ^2^=16.32; *P*<.001) among family members of HBeAg positive (21/28; 75%) than among HBeAg negative index cases (59/179; 33%). We found a positive but not strong correlation between age and HBV exposure in family members (Spearman's ρ=0.35; *P*<.001), yet we found no significant correlation between age and HBsAg seropositivity (Spearman's ρ=−0.04; *P*=.54).

**Table 1:** Proportion of HBsAg seropositivity and HBV exposure within family members (n=207) stratified by their family relationship to index case.

		Parents	Offspring	Siblings	Spouses	Total
HBsAg^a^	Positive	7 (24.1%)	16 (14.3%)	1 (14.3%)	1 (1.7%)	25 (12.1%)
	Negative	22 (75.9%)	96 (85.7%)	6 (85.7%)	58 (98.3%)	182 (87.9%)

Any HBV marker^b^	Positive	24 (82.8%)	22 (19.6%)	6 (85.7%)	28 (47.5%)	80 (38.6%)
	Negative	5 (17.2%)	90 (80.4%)	1 (14.3%)	31 (52.5%)	127 (61.4%)

HBsAg: hepatitis B surface antigen, HBeAg: hepatitis B e antigen.

a χ^2^=10.51; df=3; *P*<.01,

b χ^2^=49.33; df=3; *P*<.001.

**Table 2:** Proportion of HBsAg seropositivity and HBV exposure among family members according to sex and HBeAg seropositivity of index case.

Index case sex	HBeAg positivity	Total (n)	HBsAg positivity		HBV exposure	
			n	%	n	%
Male	Positive	20	3	15.0	13	65.0^b^
	Negative	131	6	4.6	37	28.2
	Total	151	9^b^	6.0	50^b^	33.1

Female	Positive	8	7	87.5	8	100^b^
	Negative	48	9	18.8	22	45.8
	Total	56	16^b^	28.6	30^b^	53.6

a χ^2^=17.59; *P*<.001,

b χ^2^=6.37; *P*<.02

Using univariate logistic regression analysis, significant predictors of HBsAg positivity were a female index case (odds ratio 6.31; 95% CI 2.60-15.35, *P*<.001), HBeAg positivity of an index case (odds ratio 6.07; 95% CI 2.38-15.50, *P*<.001) and being a parent of an index case (odds ratio 2.83; 95% CI 1.06-7.54, *P*<.001). When we separately analyzed groups of fathers or mothers, we found that being a father of an index case was not a significant predictor of HBsAg positivity (*P*=.99). However, being a mother of an index case was associated with HBsAg positivity (odds ratio 7.48, 95% CI 2.49-22.47, *P*<.001). Age, being a sibling, offspring or spouse of an index case were not significant predictors for HBsAg positivity among family members of index cases (*P*>.05).

For HBV exposure, significant predictors were a female index case (odds ratio 2.33, 95% CI 1.25-4.36, *P*<.01), HBeAg positivity of index case (odds ratio 6.10, 95% CI 2.46-15.17; *P*<.001), being a parent of an index case (odds ratio 10.46, 95% CI 3.79-28.83; *P*<.001), being an offspring of an index case (odds ratio 0.16, 95% CI 0.08-0.29; *P*<.001), being a sibling of an index case (odds ratio 2.33, 95% CI 1.21-86.52, *P*<.05) and age (odds ratio 1.05, 95% CI 1.03-1.07, *P*<.001), while family member sex and being a spouse of an index case were not significant predictors (*P*>.05). We used factors that were significant in the univariate regression to test their significance and level of risk in a multivariate analysis for association with HBsAg positivity and HBV exposure (Table 3). HBeAg positivity and female index case combined was a significant predictor for HBsAg positivity of family members with an odds ratio of 70.39 (95% CI 8.20-604.61; *P*<.001). Nevertheless, the combination of HBeAg positivity and female index case was not associated with HBV exposure of family members (*P*=.98).

**Table 3:** Risk factors for predicting HBsAg positivity and HBV exposure among family members of HBV chronic carriers according to multivariate logistic regression analysis.

Risk factor	HBsAg positivity (odds ratio, 95% confidence interval)	HBV exposure (odds ratio, 95% confidence interval)
Female gender of index case	11.31 (3.73-34.32, *P*<.001)	4.87 (2.21-10.72, *P*<.001)
HBeAg positivity of index case	5.56 (1.80-17.23, *P*<.005)	3.22 (1.15-9.00, *P*<.05)
Mother of index case	9.82 (2.43-39.68, *P*<.005)	3.72 (1.19-11.64, *P*<.05)

HBsAg: hepatitis B surface antigen, HBeAg: hepatitis B e antigen

## DISCUSSION

Reports from countries in our region report a prevalence of HBsAg in the general population that ranges from 2% to 10% in endemic areas with an overall HBV prevalence of 14% to 60%.^3^,^7^,^10^,^11^ Several studies that analyzed rates of HBV exposed family members of HBsAg carriers reported overall HBV prevalence rates ranging from 23% to 48%.^3^,^7^,^9^ We found comparable rates of HBV exposure in family members of chronic HBV carriers in our sample.

That the highest rate of HBV exposure and HBsAg positivity was among parents of index cases (Table 1) emphasizes the vertical route of transmission within families in our region. We have also found a relatively high number of HBV exposed cases (47.5%) among spouses of index cases, but the ratio of HBsAg positive cases among them was very low (1.7%). This reflects the fact that sexual transmission, although less predominant in our setting, usually does not end with a chronic HBsAg carrier.

It is well known that about 90% of newborns infected perinatally become chronic HBsAg carriers and most of them eventually end up with chronic hepatitis B.^12^ Vaccination programs for all infants have been established in Bosnia and Herzegovina since 2001, yet most of the adult population was not vaccinated. As a result, we have few family members (1/12 of the sample) with evidence of vaccination.

Patients positive for HBeAg are considered to be more infectious and they usually have higher titers of HBV DNA.^13^ Our figures support this since we found significantly more HBV exposed and HBsAg carriers among family members of HBeAg positive index cases.

It has been reported in previous studies that rates of HBV exposure are positively correlated with age.^14^,^15^ We have also found that each additional year of age within family members increases the chances of HBV exposure by 5% on average. This is a logical consequence of number of exposures and number of infection reservoirs within a family that increases with age.

We have demonstrated that female gender, HBeAg positivity and being a mother of an index case are strong and significant independent risk factors associated with HBV spread within the family. This is a result of a dominant mother-to-child transmission pattern, which is the most significant means of HBV spread in our sample. The presence of HBeAg facilitates this process further, rendering family members of an HBeAg positive female index carrier around 70 times more susceptible to become chronic HBV carriers themselves.

It is important to note some limitations of this study. All included index cases are from one hospital and therefore, these cases cannot necessarily represent the north-eastern region of Bosnia. The exact occurrence of HBV infection of each index case is very hard to determine and determination of the time sequence of this infection in relation to other family members is even harder. Therefore, labelling the first detected HBsAg carrier within the family is purely arbitrary and is as best a guess as possible. This methodological approach had been used in number of previous studies.^3^,^5^,^7^

All of our results indicate that the main risk factors for HBV transmission inside the family are female sex, HBeAg seropositivity in the index case and an HBsAg-positive mother in the family. This pattern of risk factors facilitates the vertical, mother-to-child route of transmission, which leads to a higher percentage of chronic HBsAg carriers, especially in younger age groups. Age appears to be another important risk factor for HBV exposure, as a direct result of the number of exposures to HBV and infection reservoirs within the family.

Children of HBeAg positive mothers are at the highest risk for becoming chronic carriers themselves and generally, the combination of female gender and HBeAg positivity dramatically increases the chances of HBV transmission within the family. Considering the intolerably low rate of HBV vaccination within family members, it is of essential importance to insist on the vaccination of this risk group in our region. This leads to the possibility that it would be a prudent strategy to investigate cost-effectiveness of prepartal screening of pregnant women for HBsAg positivity, as a first step toward a nationwide program of pregnancy screening for HBsAg that would essentially block the main route of HBV spread in our region. Also, an important addition to this strategy would be the screening and vaccination at the high school age or university age, or at the time of marriage to protect the susceptible cases born before 2001.
